# Should all breast cancer patients with four or more positive lymph nodes who underwent modified radical mastectomy be treated with postoperative radiotherapy? A population-based study

**DOI:** 10.18632/oncotarget.12260

**Published:** 2016-09-26

**Authors:** Haiyong Wang, Li Kong, Chenyue Zhang, Dawei Chen, Hui Zhu, Jinming Yu

**Affiliations:** ^1^ School of Medicine, Shandong University, Jinan, 250012, China; ^2^ Department of Radiation Oncology, Shandong Cancer Hospital affiliated to Shandong University, Shandong Academy of Medical Sciences, 250117, China; ^3^ Department of Integrative Oncology, Fudan University Shanghai Cancer Center, Shanghai 200032, China

**Keywords:** breast cancer, mastectomy, radiotherapy, positive lymph nodes, SEER

## Abstract

Postmastectomy radiotherapy (PMRT) has become a standard adjuvant postoperative therapy for breast cancer patients with four or more positive lymph nodes. However, some studies have demonstrated that some subgroups of the breast cancer patients with four or more positive lymph nodes did not benefit substantially from PMRT. Therefore, it is of great necessity to identify whether all breast cancer patients with four or more positive lymph nodes who underwent modified radical mastectomy be treated with PMRT. In our study, we first established a prognostic model using the Surveillance Epidemiology and End Results (SEER) database between 1998 and 2001. Univariate and multivariate Cox models were used to assess the prognostic factors, and five risk factors individually associated with prognosis including AJCC stage, AJCC T, Grade, ER status, PR status. Prognostic index of PMRT were defined as the number of risk factor (NRF). The NRF scores correlated well with overall survival of PMRT even if the patients were in the sub-poor prognosis group. Then the prognostic model was validated using the SEER database between 2006 and 2009, and the same results were obtained. In conclusion, different from others studies, our study demonstrated that all patients with four or more positive lymph nodes after modified radical mastectomy need to be treated with PMRT ever if the patients belonged to AJCC T4 in a poor prognosis group.

## INTRODUCTION

Breast cancer is the most frequently diagnosed cancer and the leading cause of cancer death among females worldwide, with an estimated 1.7 million incidence and 521,900 mortalities in 2012 [1]. Importantly, Breast cancer alone accounts for 25% of all cancers and 15% of all cancer-related deaths among females [1]. Lack of effective adjuvant therapies may be an important reason for its recurrence and metastasis, which would even result in the death of the patients. Postmastectomy radiation therapy (PMRT), a commonly used practice, can prevent locoregional recurrence and increase survival in breast cancer patients after definite surgery [2–4]. Previous studies have shown that PMRT therapy yielded both a substantial reduction in locoregional failure from 32% to 9% and a significant improvement in 10-year overall survival from 45% to 54% [5]. At present, National Comprehensive Cancer Network (NCCN) guidelines have intended to list PMRT as a recommended therapy for breast cancer patients with four or more positive lymph nodes. Interestingly, personalized therapy has been favored by oncologists to treat different breast cancer patients. A recent research have shown that PMRT did not have any effect on survival of these patients with four or more positive lymph nodes in a defined subgroup [6]. Therefore, whether all breast cancer patients with four or more positive lymph nodes who underwent modified radical mastectomy should be treated with PMRT needs to be tested and validated.

In the present study, using the Surveillance, Epidemiology, and End Results (SEER)-registered database, we analyzed the prognostic factors of breast cancer patients with four or more positive lymph nodes after PMRT, and answered this interesting clinical question.

## RESULTS

### Characteristics of the patients who underwent PMRT between 1998 and 2001

A total of 3972 female breast cancer patients with four or more positive lymph nodes who underwent PMRT were reported in the SEER database from 1998 to 2001. The clinical characteristics and pathological features of all the patients were summarized in Table 1. Most of the patients were diagnosed at the age of more than 40-year-old (87.9%). 51.9% of the patients were in stage IIIA according to AJCC stage, and 44.6% of the patients were in AJCC T2 stage (tumor size) and 60.6% of the patients were in N2 (lymph node stage). Almost all the patients were diagnosed in grade II and III (87.3%). In addition, 64.4%of patients were ER positive and 54.4% of patients were PR positive. The detailed statistical results were showed in Table 1.

**Table 1:** Characteristics of breast cancer patients with four or more positive lymph nodes after PMRT from SEER Database from 1998-2001

Characteristic	Number	%
**Age**		
< 40	482	12.1
≥ 40	3490	87.9
**Laterality**		
Right	2029	51.0
Left	1942	48.9
**AJCC Stage**		
II	71	1.8
IIIA	2063	51.9
IIIB	371	9.3
IIIC	1463	36.8
**AJCC T**		
T1	675	17.0
T2	1773	44.6
T3	771	19.4
T4	644	16.2
**AJCC N**		
N2	2408	60.6
N3	1560	39.3
**Grade**		
I	196	4.9
II	1305	32.9
III	2159	54.4
**ER status**		
Positive	2557	64.4
Negative	1022	25.7
**PR status**		
Positive	2159	54.4
Negative	1361	34.3

### Survival outcomes of patients between 1998 and 2001

**Figure 1:**
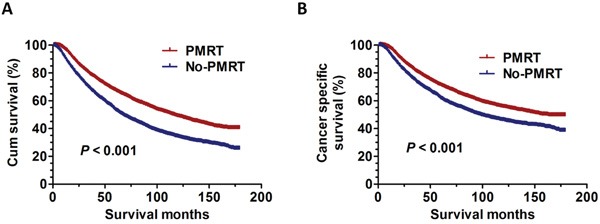
The survival curves in breast cancer patients with PMRT and No-PMRT between 1998 and 2001 **A.** The OS curves: (χ2=186.4, *P* < 0.001). **B.** The CSS curves (χ2=74.8, *P* < 0.001).

As universally acknowledged, PMRT has been a standard adjuvant postoperative therapy for patients with four or more positive lymph nodes. In our study, we identified breast cancer patients without PMRT after modified radical mastectomy to compare prognosis with those who underwent PMRT from 1998 to 2001. The clinical characteristics and pathological features of all the patients with non-PMRT were summarized in supply Table 1. As expected, the results showed that PMRT patients had better outcomes with significantly improved overall survival rate (OS) (χ2=186.4, *P* < 0.001) and cancer specific survival rate (CSS) (χ2=74.8, *P* < 0.001) compared to the non-PMRT patients (Figure 1A and 1B).

### Determination of the risk factors of patients who underwent PMRT from 1998 to 2001

The prognoses of all the patients who underwent PMRT were further analyzed using univariate and multivariate Cox proportional hazards regression analysis. The correlation between OS and various variables are summarized in Table 2. The clinicopathological features, including AJCC stage (χ2=213.6, *P* < 0.001), AJCC T (χ2=132.2, *P* < 0.001), AJCC N (χ2=107.3, *P* < 0.001), Grade (χ2=84.6, *P* < 0.001), ER status (χ2=153.9, *P* < 0.001), PR status (χ2=129.5, *P* < 0.001), were significant risk factors for OS using univariate analysis. Multivariate analysis with Cox regression was further performed and found that AJCC stage (hazard ratio [HR] 1.505; 95% confidence interval [CI] 1.308-1.732; *P* < 0.001), AJCC T (HR 1.369; 95% CI 1.245-1.505; *P* < 0.001), Grade (HR 1.226; 95% CI 1.115-1.350; *P* < 0.001), ER status (HR 1.424; 95% CI 1.258-1.613; *P* < 0.001), PR status (HR 1.213; 95% CI 1.078-1.364; *P* = 0.001) were independent prognostic factors for OS.

**Table 2:** Univariate analysis and multivariate survival analyses to evaluate the prognostic factors according to various clinicopathological variables from SEER Database from 1998-2001

Variable	Univariate analysis		Multivariate analysis	
	Log rank χ2 test	*P*	HR (95%CI)	*P*
**Age**	0.067	0.796		NI
< 40				
≥ 40				
**Laterality**	0.079	0.778		NI
Left				
Right				
**AJCC Stage**	213.644	<0.001		<0.001
II and IIIA			reference	
IIIB and IIIC			1505 (1.308-1.732)	<0.001
**AJCC T**	132.227	<0.001		<0.001
T1 and T2			reference	
T3 and T4			1.369 (1.245-1.505)	<0.001
**AJCC N**	107.269	<0.001		0.510
N2			reference	
N3			1.079 (0.945-1.232)	0.261
**Grade**	84.629	<0.001		<0.001
I and II			reference	
III			1.226 (1.115-1.350)	<0.001
**ER status**	153.872	<0.001		<0.001
Positive			reference	
Negative			1.424 (1.258-1.613)	<0.001
**PR status**	129.475	<0.001		0.001
Positive			reference	
Negative			1.213 (1.078-1.364)	0.001

NI: not included in the multivariate survival analysis.

### Establishment of the predictive model for overall survival

**Figure 2:**
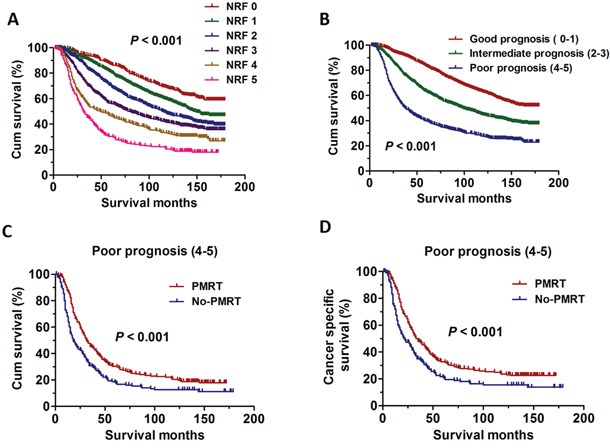
The survival curves in breast cancer patients according to different NRF scoring between 1998 and 2001 **A.** Kaplan-Meier curves for overall survival rate according to NRF scoring in six groups (*P* < 0.001). **B.** Kaplan-Meier curves for overall survival rate according to NRF scoring in three groups (*P* < 0.001). **C.** Kaplan-Meier curves for overall survival rate in breast cancer patients with PMRT and No-PMRT in poor prognosis group (χ2=15.7, *P* < 0.001). **D.** Kaplan-Meier curves for cancer specific survival rate in breast cancer patients with PMRT and No-PMRT in poor prognosis group (χ2=13.4, *P* < 0.001).

Since we have already shown that AJCC stage, AJCC T, Grade, ER status, PR status were independent prognostic factors for OS, we then construct a predictive index for the patients with four or more positive lymph nodes who underwent PMRT based on these confirmed factors. The included factors were the five risk factors, including AJCC stage, AJCC T, Grade, ER status and PR status. The predictive index was defined as the number of the risk factor (NRF). Using the NRF model, we estimated the respective OS and revealed the statistically significant differences in every group (*P* < 0.001) (Figure 2A). We then divided the patients into good prognosis (NRF 0-1), intermediate prognosis (NRF 2-3), poor prognosis (NRF 4-5) using the predictive index model. The statistically significant differences were also detected in three groups (*P* < 0.001) (Figure 2B). Besides, we try to ascertain whether the patients undergoing PMRT still have an increased OS and CSS than those without PMRT in the poor prognosis group. Interestingly, the results showed that PMRT can still significantly improve the OS (χ2=15.7, *P* < 0.001) and CSS (χ2=13.4, *P* < 0.001) of these breast cancer patients (Figure 2C–2D).

### Subgroup analysis of OS in the predictive model

**Figure 3:**
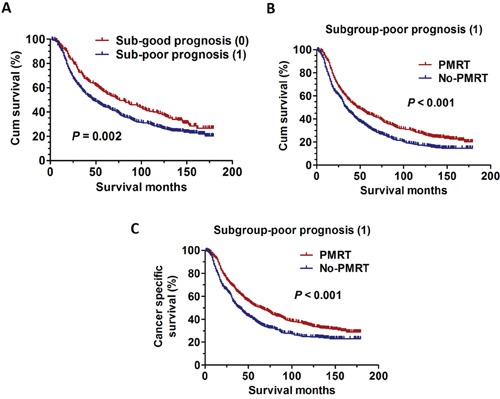
The survival curves in breast cancer patients according to different sub-groups NRF scoring between 1998 and 2001 **A.** Kaplan-Meier curves for overall survival rate according to sub-groups NRF scoring in two groups (*P* = 0.002). **B.** Kaplan-Meier curves for overall survival rate in breast cancer patients with PMRT and No-PMRT in Subgroup-poor prognosis group (χ2=18.6, *P* < 0.001). **C.** Kaplan-Meier curves for cancer specific survival rate in breast cancer patients with PMRT and No-PMRT in Subgroup-poor prognosis group (χ2=20.0, *P* < 0.001).

In the previous prediction model, AJCC Stage IIIB and IIIC, AJCC T3 and T4 were employed as variables to analyze prognosis (Table 2). Next, AJCC Stage IIIB, AJCC Stage IIIC, AJCC T3, AJCC T4 were taken as an independent variable to further analyze prognosis. Univariate and multivariate analyses were used to evaluate the relationships between OS and various variables. The results were summarized in Table 3. The results showed that AJCC T4 (χ2=9.5, *P* = 0.002) was risk factors for poor OS using univariate analysis. Multivariate analysis with Cox regression was further performed and found that AJCC T (HR 1.340; 95% CI 1.110-1.618; *P* = 0.002) was independent prognostic factors for OS. Based on these prognostic analyses, we also constructed a predictive index for these patients using the same method. The patients who were in AJCC T3-4 was quantified by the predictive index. Patients being in AJCC T3 get a point of 0 and those in AJCC T4 get a point of 1. Therefore, the NRF 0 stand for those patients with AJCC T3; the NRF 1 stand for those patients with AJCC T4; Then the patients with NRF 0 were categorized into the sub-good prognosis group. The patients with NRF 1 were categorized into the sub-poor prognosis group. The results also showed the statistically significant differences among the two groups (*P* = 0.002) (Figure 3A). In addition, we also try to ascertain whether patients undergoing PMRT still have an increased OS and CSS than those without PMRT in the sub-poor prognosis group (NRF 1). The Kaplan-Meier analyses were used to generate the survival curves and the Log Rank test was applied to analyze the differences. Interestingly, the results showed that PMRT still have survival benefit on OS (χ2=18.6, *P* < 0.001) or CSS (χ2=20.0, *P* < 0.001) for these patients (Figure 3B–3C).

**Table 3:** Univariate analysis and multivariate survival analyses to evaluate the prognostic factors according to various clinicopathological variables in sub-groups from SEER Database from 1998-2001

Variable	Univariate analysis		Multivariate analysis	
	Log rank χ2 test	*P*	HR (95%CI)	*P*
**AJCC Stage**	0.981	0.322		NI
IIIB				
IIIC				
**AJCC T**	9.463	0.002		0.002
T3			reference	
T4			1.340 (1.110-1.618)	0.002

NI: not included in the multivariate survival analysis.

### Validation of the predictive model for overall survival

**Figure 4:**
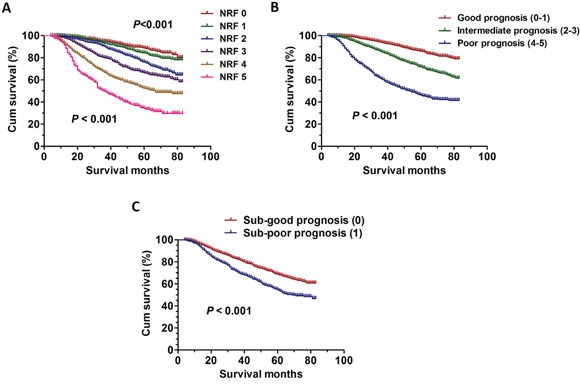
The survival curves in breast cancer patients according to different NRF scoring between 2006 and 2009 **A.** Kaplan-Meier curves for overall survival rate according to NRF scoring in six groups (*P* < 0.001). **B.** Kaplan-Meier curves for overall survival rate according to NRF scoring in three groups (*P* < 0.001). **C.** Kaplan-Meier curves for overall survival rate according to NRF scoring in three sub-groups (*P* < 0.001).

We have established a predictive index for the patients with four or more positive lymph nodes who underwent PMRT. Next, we identified another group of breast cancer patients with four or more positive lymph nodes who underwent PMRT reported in the SEER database from 2006 to 2009. The clinical characteristics and pathological features of all the patients were summarized in supply Table 2. Consistent with the prediction model, the results also showed the statistically significant differences among the groups using the same predictive index model (*P* < 0.001) (Figure 4A). In addition, the results also showed that the statistically significant differences were detected in the good prognosis (NRF 0-1), intermediate prognosis (NRF 2-3), poor prognosis (NRF 4-5) group (*P* < 0.001) (Figure 4B). Then, the subgroup analyses were further performed and the statistically significant differences were also detected in Sub-good prognosis (NRF 0), Sub-intermediate prognosis (NRF 1) (*P* < 0.001) (Figure 4C).

### Whether patients in the sub-poor group can benefit from PMRT: the validation

**Figure 5:**
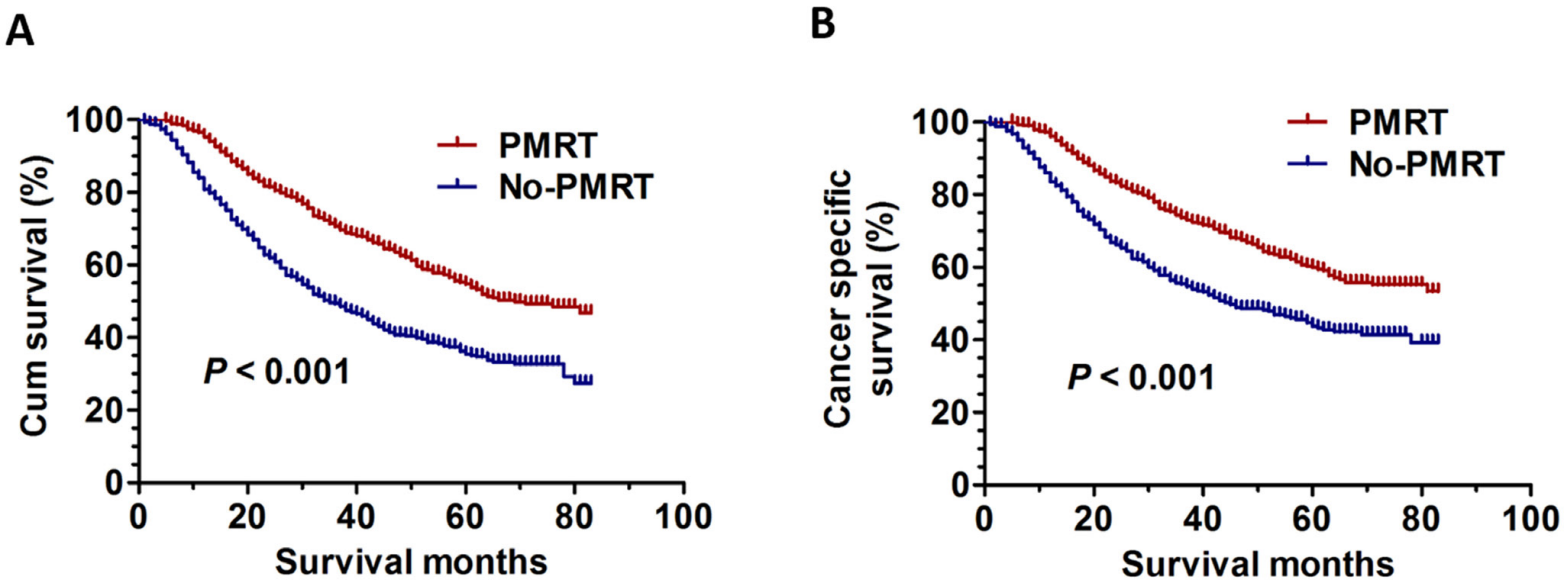
The survival curves in breast cancer patients with PMRT and No-PMRT in Sub-poor prognosis group **A.** Kaplan-Meier curves for overall survival rate in breast cancer patients with PMRT and No-PMRT in Sub-poor prognosis group between 2006 and 2009 (χ2=62.1, *P* < 0.001). **B.** Kaplan-Meier curves for cancer specific survival rate in breast cancer patients with PMRT and No-PMRT in Sub-poor prognosis group between 2006 and 2009 (χ2=42.5, *P* < 0.001).

We have shown that the breast cancer patients with four or more positive lymph nodes after modified radical mastectomy can benefit from the PMRT even if the patients were in AJCC T4 (Figure 3B–3C). Next, according to the same standards, we identified the patients with AJCC T4 in the SEER database from 2006 to 2009. The Kaplan-Meier analyses were used to generate the survival curves and the Log Rank test was applied to analyze the differences. As expected, the results also showed PMRT still have some survival benefit on OS (χ2=62.1, *P* < 0.001) or CSS (χ2=42.5, *P* < 0.001) for these patients (Figure 5A–5B).

## DISCUSSION

In the present study, we concluded that the breast cancer patients with four or more positive lymph nodes aftermodified radical mastectomy can benefit from the PMRT even if the patients were in AJCC T4.

In fact, early randomized trials of breast cancer with PMRT did not prove the improvement in overall survival and just demonstrated that the risk of locoregional recurrence was reduced [8–10]. With the popularity of systemic therapy and the development of radiotherapy, several studies have demonstrated that the patients of breast cancer may benefit from the administration of PMRT. In a Danish trial of premenopausal patients, The frequency of locoregional recurrence alone or with distant metastases was 9% among the women who received radiotherapy plus systemic chemotherapy and 32% among those who received systemic chemotherapy alone; In addition, the probability of survival free of disease and the overall survival after 10 years was 48% and 54% among the women assigned to radiotherapy systemic chemotherapy and 34% and 45% among those treated only with systemic chemotherapy; Importantly, multivariate analysis demonstrated that PMRT significantly improved disease-free survival and overall survival, irrespective of tumor size, the number of positive nodes, or the histopathological grade [5]. In a Danish trial of postmenopausal patients, the rate of locoregional recurrence was 8% in the radiotherapy plus tamoxifen group and 35% in the tamoxifen group; Disease-free survival was 36% in the radiotherapy plus tamoxifen group and 24% in the tamoxifen alone group; Overall survival was also higher in the radiotherapy group (385 vs 434 deaths; survival 45 vs 36% at 10 years) [11]. Importantly, several other studies have also demonstrated PMRT does indeed decrease the mortality and locoregional recurrence [12–14]. The most influential study of these was a meta-analysis conducted by the Early Breast Cancer Trialists’ Collaborative Group (EBCTCG). This study included 8500 with mastectomy, axillary clearance, and node-positive disease in trials of radiotherapy, with similar absolute gains from radiotherapy; 5-year local recurrence risks 6% versus 23% (reduction 17%), and 15-year breast cancer mortality risks 54.7% versus 60.1% (reduction 5.4%) [15]. However, some limitation still exist in these trials. For example, the median number of lymph nodes removed was 7 in the Danish 82b and 82c trials. Interestingly, the status and dissection of axillary lymph nodes especially with regard to the number of removed lymph nodes has a critical role in breast cancer patients, and the number of negative lymph nodes can affect the prognosis of breast cancer patients [6, 16–19]. Indeed, the breast cancer patients after mastectomy with 1 to 3 positive lymph nodes who haven't received PMRT has been demonstrated have lower rates of locoregional recurrence than observed in the Danish and other trials [20–22]. Therefore, at present, consensus guidelines have intended to recommend PMRT for patients who have ≥ 4 involved positive lymph nodes [23–26]. Importantly, the National Comprehensive Cancer Network has recommend that the breast cancer patients with ≥ 4 positive lymph nodes should perform PMRT to the chest wall, supraclavicular, and infraclavicular regions and radiation to the internal mammary region.

Similar to these studies, using the SEER database, we screened the breast cancer patients with four or more positive lymph nodes after PMRT between 1998 and 2001. Our results also showed that PMRT significantly improved patient prognosis on OS and CSS.

At present, individualized treatments are a trend for oncologists to determine different treatment options. Studies have demonstrated that the patients with four or more positive lymph nodes who did not receive PMRT have the local recurrence rate from 11.9 to 59% [27, 28]. That means about 40% of these patients do not benefit from PMRT. Interestingly, a study from China has demonstrated that PMRT did not have any effect on the survival of breast cancer patients with four or more positive lymph nodes if the patients have more than 12 negative lymph nodes [6]. Therefore, it is very important to identify those patients with four or more positive lymph nodes that are not likely to benefit from PMRT. Also, we aim to ascertain whether all breast cancer patients with four or more positive lymph nodes should be treated with PMRT.

Firstly, we screened the breast cancer patients with four or more positive nodes who underwent modified radical mastectomy using the SEER database between 1998 and 2001. Univariate and Multivariate analysis was used and showed that AJCC stage, AJCC T, Grade, ER status, PR status were independent prognostic factors for OS. Then we construct a predictive index for these patients. The predictive index was defined as NRF refer to other study [29]. As expected, the overall survival rate was lower if the patients undergoing PMRT are with the higher NRF score. Interestingly, even if the patient has the highest NRF score, PMRT still improves the survival of patients on OS and CSS. In our study, we took AJCC stage IIIB and IIIC, AJCC T3 and T4as variables respectively. In order to identify high-risk patients, we next took AJCC stage IIIB, AJCC stage IIIC, AJCC T3, AJCC T4 as independent variables respectively. Univariate and Multivariate analysis was also used and showed that AJCC T3 and T4, were independent prognostic factors for OS. In addition, the patients with AJCC T4 have the worst prognosis. Surprisingly, we found that PMRT still have some survival benefit on OS and CSS in patients with AJCC T4.

As we all know, radiation technology has been advancing over the past few years. The accumulation of radiation experience and the development of technology also play important roles in improving prognosis of patients. Secondly, we screened the breast cancer patients with four or more positive lymph nodes after PMRT and validated the previous prognostic model in recent few years between 2006 and 2009. The results also showed the overall survival rate was lower if the patients underwent PMRT with the higher NRF score, and PMRT also have survival benefit on OS and CSS in patients with AJCC T4.

This study also has several limitations. Firstly, some studies have showed that the systemic therapy may bring some substantial survival benefits for PMRT [30–32]. However, due to the absence of information on chemotherapy or targeted therapy included in the SEER database, its effect on survival could not be evaluated. Secondly, this study is the non-randomized study and the intrinsic defects exist in any retrospective study. Thirdly, some factors that obviously influence the prognosis of breast cancer patients, such as the number of negative lymph nodes, Her2 status, were not included in our study. Given the limitations of the SEER dataset. Therefore, future prospective studies from different countries and regions are needed to further confirm these results.

In conclusion, our study demonstrated that all patients with four or more positive lymph nodes after modified radical mastectomy need be treated with PMRT even the patients with AJCC T4.

## MATERIALS AND METHODS

### Patient selection

The SEER Cancer Statistics Review (http://seer.cancer.gov/data/citation.html) is published annually by the Data Analysis and Interpretation Branch of the National Cancer Institute, MD, USA. A total of 18 population-based cancer registries in the United States were included in the current SEER database [7]. The SEER*Stat software (SEER*Stat 8.2.1) was used to identify the appropriate patients. Using this software, we first screened breast cancer patients after PMRT between 1998 and 2001 to determine the risk factors. Then we validated these risk factors between 2006 and 2009 and ultimately identify the breast cancer patients who are not likely to benefit from PMRT. The included patients should meet the following criteria: the diagnosis was confirmed microscopically, and they should be female with the confirmed age, active follow-up and only one primary tumor. In addition, the patients should be those who have received modified radical mastectomy, with at least four positive lymph nodes removed. Patients with benign or borderline tumors, unknown age, unknown cause of death, and unknown survival months were all excluded.

### Ethics statement

This study was mainly based on the SEER database and was conducted in compliance with the Helsinki Declaration. We obtained permission to access the files of SEER program research data and the reference number is 11824-Nov2014. The informed consent was not required because personal identifying information was not involved. This study was approved by the ethics committee of the Shandong Cancer Hospital affiliated to Shandong University.

### Statistical analysis

For all the patients, the following variables were analyzed: Age, Laterality, AJCC stage, AJCC T, AJCC N, Grade, ER status, PR status. In addition, the OS and CSS were regarded as the primary endpoint of this study and extracted from the SEER database. χ^2^ tests were used to compare the patient baseline characteristics. The Kaplan-Meier analyses were used to generate the survival curves and the Log Rank test was applied to analyze the differences among the curves. Comparative risks of mortality were evaluated using univariate and multivariate Cox proportional hazards regression models. All statistical tests were two-sided, and a *P* < 0.05 was considered statistically significant. The statistical software SPSS 18.0 (SPSS, IL, Chicago) was used for all data analysis.

## SUPPLEMENTARY TABLES


