# Supplementary material for: Should all breast cancer patients with four or more positive lymph nodes who underwent modified radical mastectomy be treated with postoperative radiotherapy? A population-based study
Source: Oncotarget. 2016 Sep 26;7(46):75492–502. doi: 10.18632/oncotarget.12260 (PMC5342755; doi:10.18632/oncotarget.12260)
Supplement: Supplementary file 1 [file oncotarget-07-75492-s001.pdf]

Should all breast cancer patients with four or more positive lymph nodes who underwent modified radical mastectomy be treated with postoperative radiotherapy? A population-based study

SUPPLEMENTARY TABLES

Supplementary Table S1: Characteristics of breast cancer patients with four or more positive lymph nodes without PMRT from SEER Database from 1998-2001

Characteristic	Number	%
**Age**		
< 40	290	9.5
≥ 40	2749	90.5
**Laterality**		
Right	1536	50.5
Left	1498	49.3
**AJCC Stage**		
II	123	4.1
IIIA	1612	53.0
IIIB	235	7.7
IIIC	1069	35.2
**AJCC T**		
T1	530	17.4
T2	1421	46.8
T3	554	18.2
T4	442	14.5
**AJCC N**		
N2	1967	64.7
N3	1069	35.2
**Grade**		
I	141	4.6
II	1056	34.7
III	1593	52.4
**ER status**		
Positive	1673	55.1
Negative	772	25.4
**PR status**		
Positive	1346	44.2
Negative	1016	33.4

Supplementary Table S2: Characteristics of breast cancer patients with four or more positive lymph nodes with PMRT or without PMRT from SEER Database from 2006-2009

Variable	PMRT		No-PMRT	
	Number	%	Number	%
**Age**				
< 40	521	10.6	225	6.9
≥ 40	4393	89.4	3029	93.1
**Laterality**				
Left	2466	50.2	1631	50.1
Right	2448	49.8	1623	49.9
**AJCC Stage**				
II and IIIA	2746	55.9	1795	55.2
IIIB and IIIC	2168	44.1	1459	44.8
**AJCC T**				
T1 and T2	2942	59.9	2040	62.7
T3 and T4	1972	40.1	1214	37.3
**AJCC N**				
N2	3222	65.6	2084	64.0
N3	1692	34.4	1170	36.0
**Grade**				
I and II	2375	48.3	1484	45.6
III	2539	51.7	1770	36.0
**ER status**				
Positive	3713	75.6	2320	71.3
Negative	1201	24.4	922	28.3
**PR status**				
Positive	3244	66.0	1932	59.4
Negative	440	9.0	1322	40.6
